# Morpho-functional comparison of differentiation protocols to create iPSC-derived cardiomyocytes

**DOI:** 10.1242/bio.059016

**Published:** 2022-02-23

**Authors:** Aleksandra Nijak, Eline Simons, Bert Vandendriessche, Dieter Van de Sande, Erik Fransen, Ewa Sieliwończyk, Ilse Van Gucht, Emeline Van Craenenbroeck, Johan Saenen, Hein Heidbuchel, Peter Ponsaerts, Alain J. Labro, Dirk Snyders, Winnok De Vos, Dorien Schepers, Maaike Alaerts, Bart L. Loeys

**Affiliations:** 1 Center of Medical Genetics, Faculty of Medicine and Health Sciences, University of Antwerp & Antwerp University Hospital, Antwerp 2650, Belgium; 2 Laboratory of Molecular Biophysics, Cellular and Network Excitability, Department of Biomedical Sciences, University of Antwerp, Antwerp 2610, Belgium; 3 StatUa Center of Statistics, University of Antwerp 2650, Antwerp, Belgium; 4 Department of Cardiology, Faculty of Medicine and Health Sciences, University of Antwerp and Antwerp University Hospital, Antwerp 2650, Belgium; 5 Laboratory of Experimental Hematology, Vaccine & Infectious Disease Institute, Department of Biomedical Sciences, University of Antwerp, Antwerp 2610, Belgium; 6 Department of Basic and Applied Medical Sciences, Faculty of Medicine and Health Sciences, Ghent University, Ghent 9000, Belgium; 7 Laboratory of Cell Biology and Histology, Faculty of Veterinary Sciences, University of Antwerp, Antwerp 2610, Belgium; 8 Department of Human Genetics, Radboud University Medical Centre, Nijmegen 6525, The Netherlands

**Keywords:** iPSC-CMs, Cardiomyocyte differentiation, Arrhythmia modelling

## Abstract

Cardiomyocytes derived from induced pluripotent stem cells (iPSC-CMs) offer an attractive platform for cardiovascular research. Patient-specific iPSC-CMs are very useful for studying disease development, and bear potential for disease diagnostics, prognosis evaluation and development of personalized treatment. Several monolayer-based serum-free protocols have been described for the differentiation of iPSCs into cardiomyocytes, but data on their performance are scarce. In this study, we evaluated two protocols that are based on temporal modulation of the Wnt/β-catenin pathway for iPSC-CM differentiation from four iPSC lines, including two control individuals and two patients carrying an *SCN5A* mutation. The *SCN5A* gene encodes the cardiac voltage-gated sodium channel (Na_v_1.5) and loss-of-function mutations can cause the cardiac arrhythmia Brugada syndrome. We performed molecular characterization of the obtained iPSC-CMs by immunostaining for cardiac specific markers and by expression analysis of selected cardiac structural and ionic channel protein-encoding genes with qPCR. We also investigated cell growth morphology, contractility and survival of the iPSC-CMs after dissociation. Finally, we performed electrophysiological characterization of the cells, focusing on the action potential (AP) and calcium transient (CT) characteristics using patch-clamping and optical imaging, respectively. Based on our comprehensive morpho-functional analysis, we concluded that both tested protocols result in a high percentage of contracting CMs. Moreover, they showed acceptable survival and cell quality after dissociation (>50% of cells with a smooth cell membrane, possible to seal during patch-clamping). Both protocols generated cells presenting with typical iPSC-CM AP and CT characteristics, although one protocol (that involves sequential addition of CHIR99021 and Wnt-C59) rendered iPSC-CMs, which were more accessible for patch-clamp and calcium transient experiments and showed an expression pattern of cardiac-specific markers more similar to this observed in human heart left ventricle samples.

## Abstract (precis)

**Summary:** In this study, we evaluated two protocols that are based on temporal modulation of the Wnt/β -catenin pathway for iPSC-CM differentiation from four iPSC lines. We show that both protocols were successful in the generation of contracting iPSC-CMs. However, one of the tested protocols rendered cells that were more accessible for patch-clamp experiments and showed an expression pattern of cardiac-specific markers more similar to this of human heart left ventricle samples.

## INTRODUCTION

**Fig. 1.**
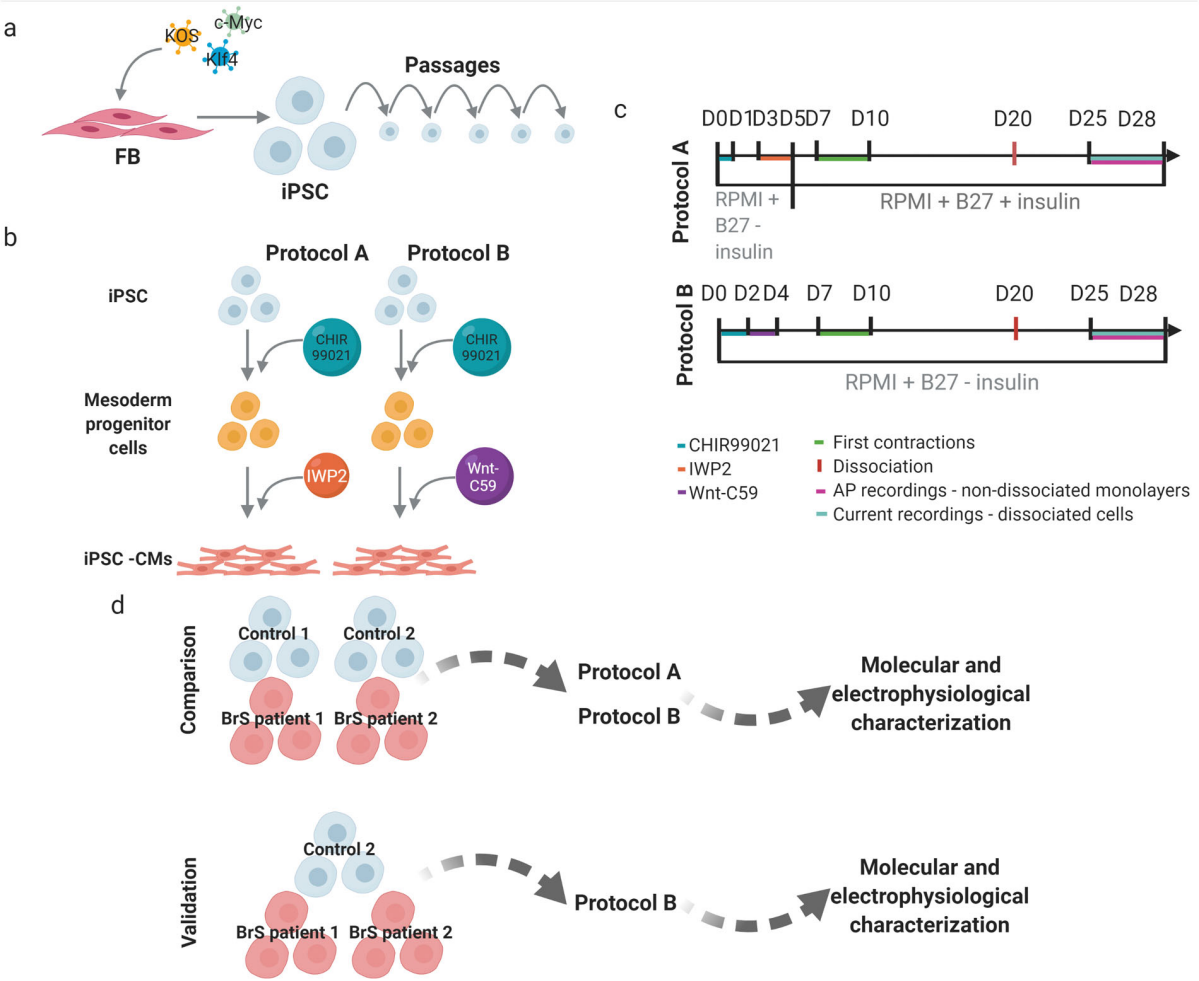
**Schematic presentation of the workflow and experimental design.** (A) Reprogramming of the fibroblasts into iPSC. (B) Derivation of cardiomyocytes. (C) Timelines for the tested differentiation protocols with addition of small molecules, first observed contractions, time point of dissociation of the cells as well as time window for patch-clamping experiments indicated. (D) Experimental design used for comparison assessment of the selected differentiation protocols (created with biorender.com, August 2021).

A major challenge for contemporary cardiac research is acquisition of tissue specific cells for *in vitro* modelling. As it is not routine to obtain cardiomyocytes from a patient, induced pluripotent stem cells (iPSCs) serve as an attractive alternative source for derivation of cardiomyocytes (CMs, iPSC-CMs; Fig. 1A). The first attempts to differentiate pluripotent stem cells into CMs employed a suspension culture of human embryonic stem cells (hESCs) in medium containing serum, to form so-called embryoid bodies (EB) (Kehat et al., 2001). However, this approach resulted in low numbers of hESC-CM (5–10%). Later on, the suspension protocol was improved with the use of defined serum-free media, supplemented with growth factors involved in normal human embryological heart development, like activin A, bone morphogenetic proteins (BMPs), fibroblast growth factor 2 (FGF2), vascular endothelial growth factor (VEGF), Dickkopf-related protein (DKK) and Wnt agonists and antagonists (Zhang et al., 2012; Yang et al., 2008; Burridge et al., 2011; Lian et al., 2012; Minami et al., 2012). Although those protocols showed improved CM yield to about 40–90%, the 3D EB clusters hamper even diffusion of the growth factors in the cell culture, having an impact on reproducibility of the procedure (Correia et al., 2014). To overcome the obstacles of suspension protocols, an application of monolayer cell culture with addition of the aforementioned growth factors was proposed. Multiple monolayer-based differentiation approaches have been reported, with high efficiency (80–99% estimated based on the proportion of troponin T or cardiac α-actinin positive cells in flow cytometric measurements) and reproducibility tested in multiple laboratories (Bhattacharya et al., 2014; D'Amour et al., 2005; Lian et al., 2013a,b; Burridge et al., 2014; Ren et al., 2011).

Based on the fact that iPSC-CMs express cardiac specific structural and ion channel proteins, they can be used for modelling cardiac activity *in vitro*. The first reported use of iPSC-CMs as a model for arrhythmias concerned long QT syndrome (LQTS) (Moretti et al., 2010). The study showed that iPSC-CMs enable the investigation of an arrhythmic phenotype, based on differences in action potential duration (APD) and decrease in potassium current density when patient iPSC-CMs were compared to control iPSC-CMs. Further on, iPSC-CM models for other inherited cardiac arrhythmias, including Brugada syndrome (BrS) have been successfully characterized (Yazawa et al., 2011; Jung et al., 2012; Kim et al., 2013; Cerrone et al., 2014; Hwang et al., 2015; Nijak et al., 2021). Electrophysiological characterization of these models is typically performed using patch-clamping experiments, however, alternate more high-throughput data acquisition techniques such as intracellular Ca^2+^ transient (CT) recordings using optical dyes or field potential recordings have also been successfully applied (Hwang et al., 2015; Nijak et al., 2021).

To date, available reports on comparison of differentiation protocols focus on performance of 3D versus monolayer-based approaches from a single control donor. The aim of this report is to compare two selected differentiation protocols based on (i) their overall performance in generation of iPSC-CMs from iPSC clones from several donors and (ii) the morpho-functional characteristics of the created iPSC-CMs. We compared two monolayer-based serum-free differentiation protocols, which adopt a two-step procedure: mesoderm induction through activation of the Wnt/β-catenin pathway using medium supplemented with a GSK3 inhibitor small molecule – CHIR99021, followed by cardiac-fate determination through inhibition of the canonical Wnt pathway using media supplemented with IWP2 (protocol A; Lian et al., 2013a) and Wnt-C59 (protocol B; Burridge et al., 2014) (Fig. 1B). In order to select the best performing differentiation protocol, we followed the subsequent experimental outline: one iPSC clone from four individuals (Control 1 and 2, BrS patient 1 and 2) was used, each differentiated in four or six replicates (one well on a six-well plate=one replicate) using protocol A and B (Fig. 1D). We investigated growth morphology, contracting area, survival after dissociation, expression of cardiac specific proteins and mRNA expression level of cardiac structural and ion channel proteins. As in subsequent studies we aim to model cardiac arrhythmias, for the evaluation of the best performing protocol we looked at electrophysiological properties of the obtained cells. In patch-clamp experiments we characterized action potential (AP) properties, including APD, action potential amplitude (APA), resting membrane potential (RMP) and beating rate (beats per minute, BPM). In addition, we used optical dyes to characterize CT properties: calcium transient duration (CTD), time of rise and decay and beating rate. In this report we do not address *SCN5A* expression, nor sodium currents and AP upstroke velocity, since we expect these to be different in patient iPSC-CMs based on the mutation status of the selected patient donors and these are beyond the scope of this paper.

## RESULTS

### Reprogrammed iPSC clones express pluripotency markers and show appropriate iPSC morphology

We derived 10–12 iPSC clones from each individual fibroblast line and validated three of them. All of the validated clones selected for the experiments presented good iPSC colony morphology (bright small round cells forming compact colonies with smooth, defined edges), showed expected expression of all of the tested pluripotency markers and presented no relevant novel genomic aberrations (CNVs>100 kb, affecting genes involved in cardiomyocyte function or development) after the reprogramming process (Fig. S1 and Table S1).

### Protocol comparison: iPSC-CMs obtained with protocol B show higher similarity in transcript expression in comparison to ventricular tissue, and show less 3D clumps morphology

The first observable features of differentiating iPSC-CMs are changes in cell morphology, which in the end lead to the development of spontaneously contracting cells. We collected light microscopy images of contracting cell cultures and observed three types of growth morphology: full flat monolayer, patches of monolayer and 3D cell clumps. Representative examples of growth morphology are presented in Table 1. Both protocols generated contracting CMs from Control 1 and 2 and BrS patient 2 iPSCs, while for BrS patient 1 only protocol B was successful (Table 1). We noted morphology of 3D clumps in iPSC-CMs generated with protocol A but not with protocol B. Overall, protocol A generated contracting cells in 7 out of 18 and protocol B in 15 out of 18 replicates, with on average larger contracting area in iPSC-CMs generated with protocol B (11±1% and 33±5.6% average contracting area for protocols A and B, respectively).

**Table 1.**
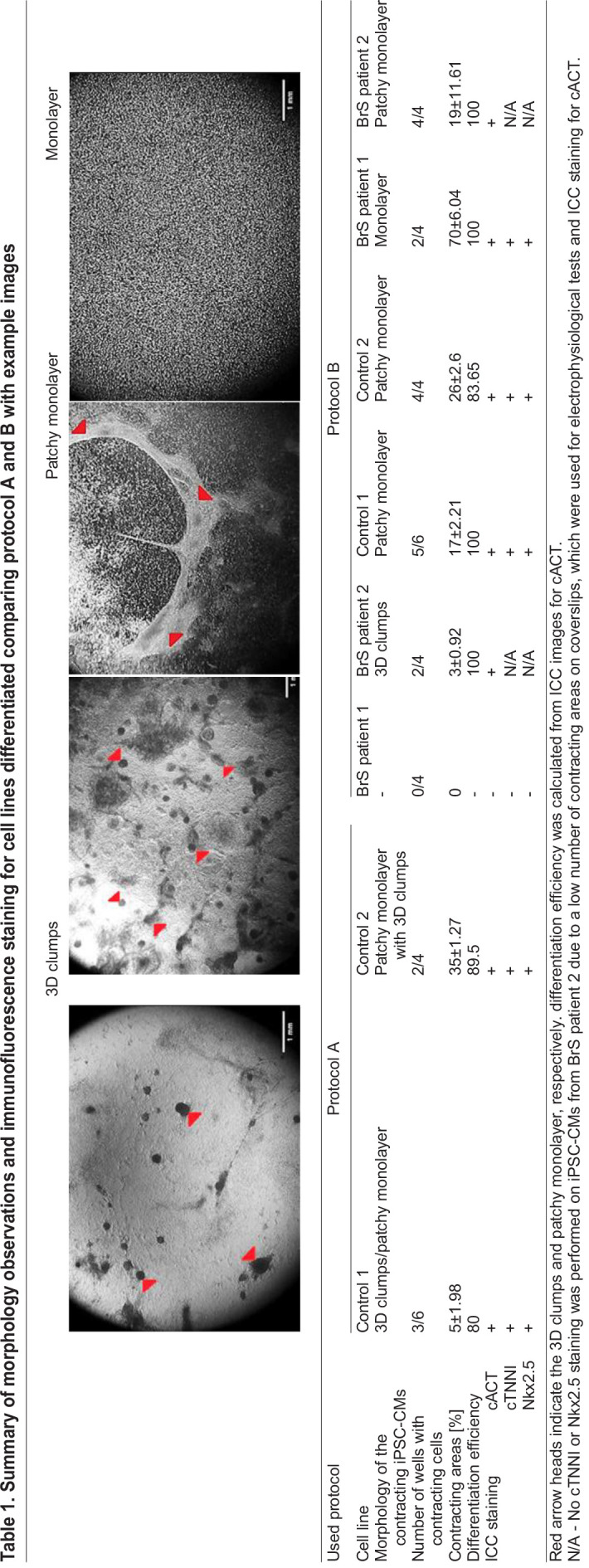
Summary of morphology observations and immunofluorescence staining for cell lines differentiated comparing protocol A and B with example images

**Fig. 2.**
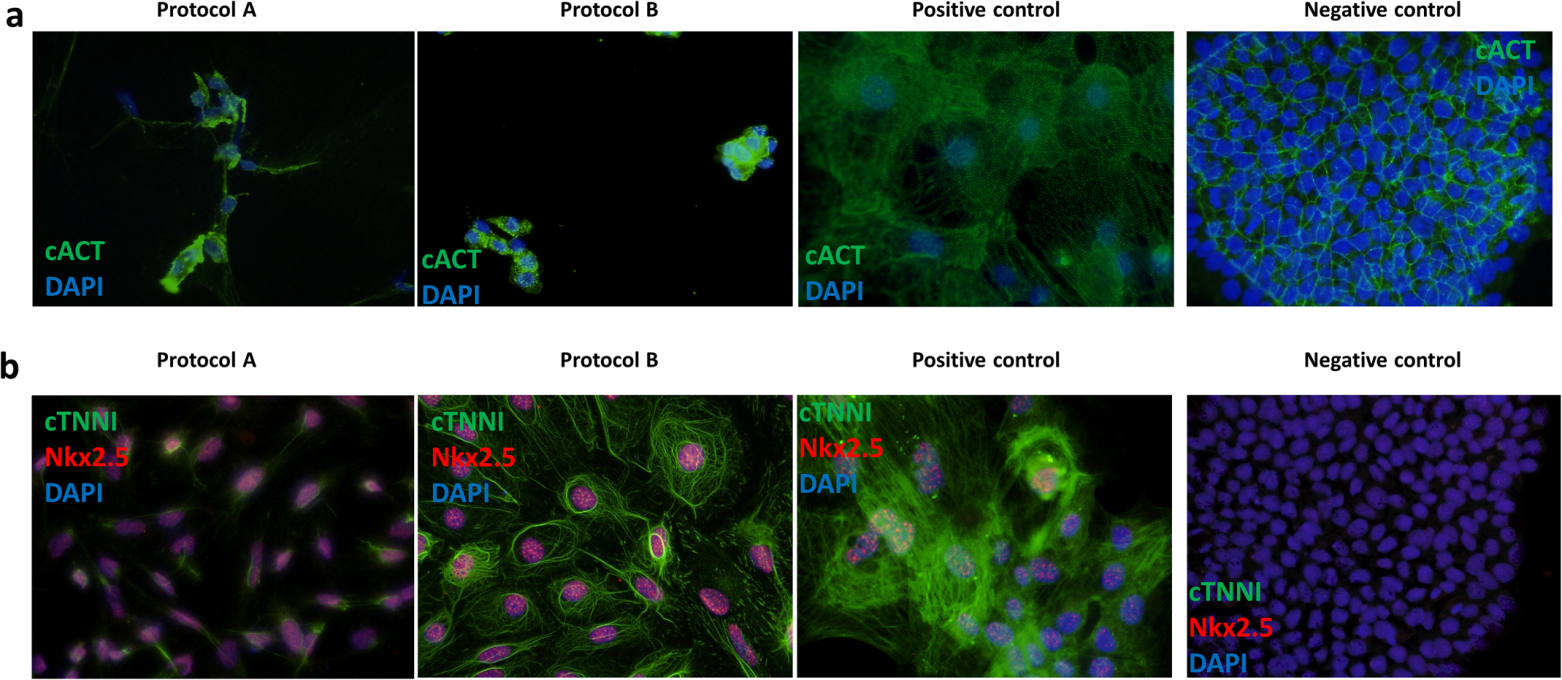
**Representative immunofluorescence staining of cardiac markers and patch-clamping experiment results for iPSC-CMs in the comparison of protocol A and B.** (A) Staining of cardiac α-actinin (cACT) on dissociated and (B) cardiac troponin I (cTNNI) and Nkx2.5 on non-dissociated iPSC-CMs. Nuclei were visualized using DAPI. A positive (commercial iPSC-CMs) and negative (iPSC) control staining results are shown for both tested antibody combinations.

**Fig. 3.**
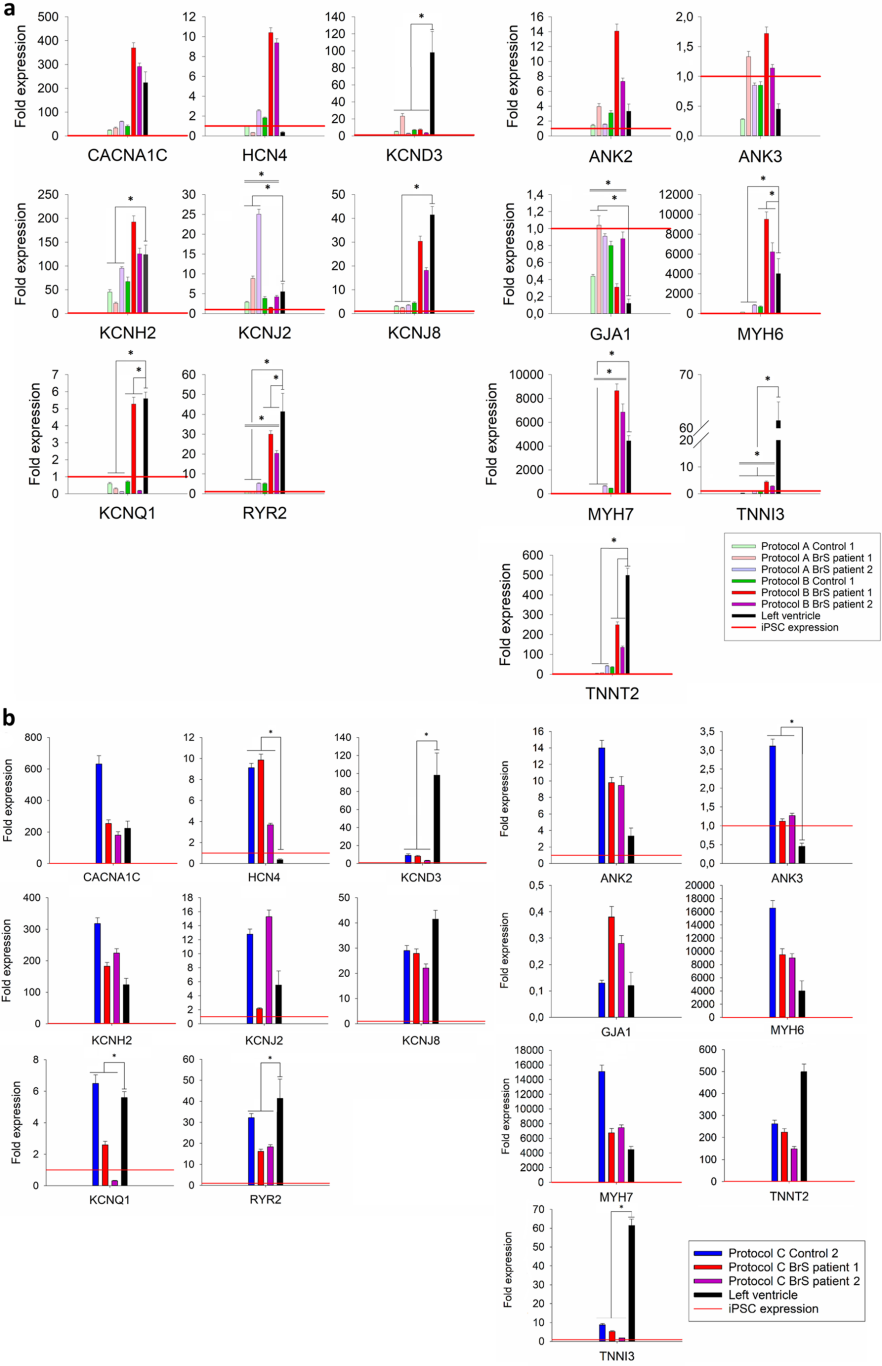
**Transcript levels of selected cardiac ion channel and structural genes in generated iPSC-CMs as fold expression compared to iPSCs.** (A) Relative expression levels of tested cardiac markers for cells differentiated with protocols A and B: in light green Control 1, in light red BrS patient 1, in light purple BrS patient 2; protocol B: in green Control 1, in red BrS patient 1, in purple BrS patient 2. (B) Relative expression levels of tested cardiac markers for: Control 2 presented in blue, BrS patient 1 in red and BrS patient 2 in purple, obtained in validation experiment of protocol B. The graphs show the average ±s.d. values from triplicates obtained from the tested samples. In each panel, measured expression levels of the analyzed cardiac markers in left ventricle tissue of a healthy donor (in black) are indicated next to the expression measured in the analyzed iPSC-CM samples. Relative transcript expression level in iPSC is indicated with red horizontal line for each of the tested markers (fold expression=1). Statistically significant differences in relative transcript expression between the tested protocols and in relation to LV tissue are indicated (respectively) with a double or a single line and a * above the graphs. Calculated *P*-values for each tested marker between two groups (protocol A versus protocol B) and in relation to the LV tissue are listed in Table S3.

All of the generated contracting iPSC-CMs showed expression of all tested cardiac protein markers (cACT, cTNNI and Nkx2.5) in ICC staining for protocol A and B (Fig. 2A,B). The iPSC-CMs differentiated using protocol A showed less organization of cTNNI, where generated cells showed cTNNI expression mainly around the nucleus and not presenting cytoskeletal structures visible in the cells obtained with protocol B (Fig. 2B; Fig. S2). Average differentiation efficiency calculated from ICC staining of cACT was similar for iPSC-CMs generated using both protocols (protocol A: 90.2±4.1%; protocol B: 95.9±4.1%) (Fig. 2A, Table 1; Table S2), although for BrS patient 1 protocol A did not produce CMs. We evaluated mRNA levels of selected cardiac markers in iPSC-CMs for both protocols (except for control 2 for which insufficient RNA concentration was obtained). Results showed significant differences in the expression levels for five of the markers (*GJA1*, *KCNJ2*, *MYH7*, *RYR2*, *TNNI3*) between the used protocols (Fig. 3A; Table S3). We observed significantly lower relative expression of *KCND3*, *KCNQ1*, *RYR2*, *TNNI3* and *TNNT2* in iPSC-CMs obtained using both protocols, in comparison to left ventricular (LV) cardiac tissue (Fig. 3A; Fig. S3A, Table S3) as a reference. Additionally, cells generated with protocol A showed significantly lower relative expression of *KCNH2*, *KCNJ8*, *MYH6* and *MYH7*, as well as higher relative expression of *KCNJ2* and *GJA1*, while cells generated with protocol B showed significantly higher relative expression of *MYH6* compared with the reference tissue. Although both protocol A and B show differences in expression compared to LV tissue, protocol B showed more similar expression levels of each of the tested genes in comparison to LV tissue (Fig. S3a).

An important feature of the created iPSC-CMs is proper survival after dissociation to allow single-cell patch-clamp recordings, especially required for characterization of specific ionic currents. After dissociation we saw that cells obtained with both protocols showed sufficient survival (>50% of single cells attached to the cover slips were round with smooth cell membrane). For AP characterization, we performed AP recordings on a non-dissociated monolayer, as these reflect a more physiologically relevant condition (Van de Sande et al., 2021). We were able to obtain AP recordings for three of the differentiated cell lines (Control 2, BrS patient 1 and 2), however their quality impacted the number of recordings which could be analyzed (Table S4). Based on the numbers of recordings per cell line (average 6.7 for protocol A and 10.7 for protocol B; Table S4), the cells generated with protocol B were more approachable (flatter and more elongated) for patch-clamp experiments than those obtained with protocol A. Due to inappropriate RMP (above −50 mV, while physiological RMP ranges from −80 to −90 mV; and iPSC-CMs RMP ranges from −40 to −70 mV), absence of spontaneous activity and/or declining seal quality (due to vivid contractions), we obtained a sufficient number of good quality AP recordings from iPSC-CMs created only by protocol B (BrS patient 1 and 2, *n*=4 and 2, respectively) (Table S4).

In conclusion, only the application of protocol B led to successful differentiation of all four lines into contracting iPSC-CMs. Protocol A resulted predominantly in 3D morphology, which was not observed in cells obtained with protocol B. Both protocols showed similar differentiation efficiency based on ICC staining, and generated iPSC-CMs expressing all the tested cardiomyocyte-specific markers at the RNA as well as the protein level. However, the expression pattern of cells generated with protocol B resembled that of adult left ventricle more closely and they presented better expression and organization of cTNNI in ICC staining. Finally, the cells generated by protocol B showed a more physiological flatter and more elongated morphology, and were more amenable for patch-clamping, while 3D clumps observed in iPSC-CMs obtained with protocol A impaired their electrophysiological characterization. As such, for future experiments, we opted to focus on further validation of protocol B.

### Protocol validation: iPSC-CMs generated with protocol B are suitable for patch-clamping and intracellular Ca^2+^ imaging

Since protocol B was the best performing protocol, we decided to perform a more detailed electrophysiological validation of the generated iPSC-CMs. All tested cell lines differentiated in a contracting/patchy monolayer in all of the replicates for Control 2, three out of six replicates for BrS patient 1 and two out of six replicates for BrS patient 2 (Table 2). Average contracting areas for Control 2 amounted to 97±0.01%, for BrS patient 1 to 19±10.5% and BrS patient 2 to 80±2.2% (Table 2).

**Table 2.**
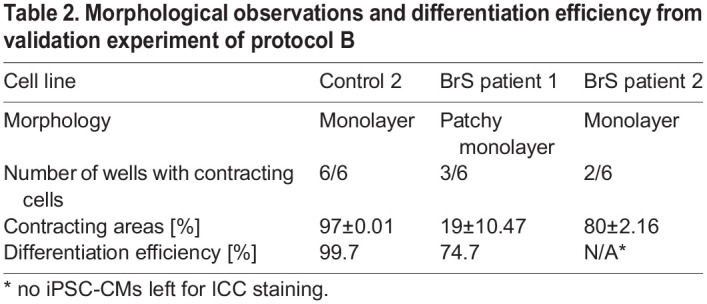
Morphological observations and differentiation efficiency from validation experiment of protocol B

**Fig. 4.**
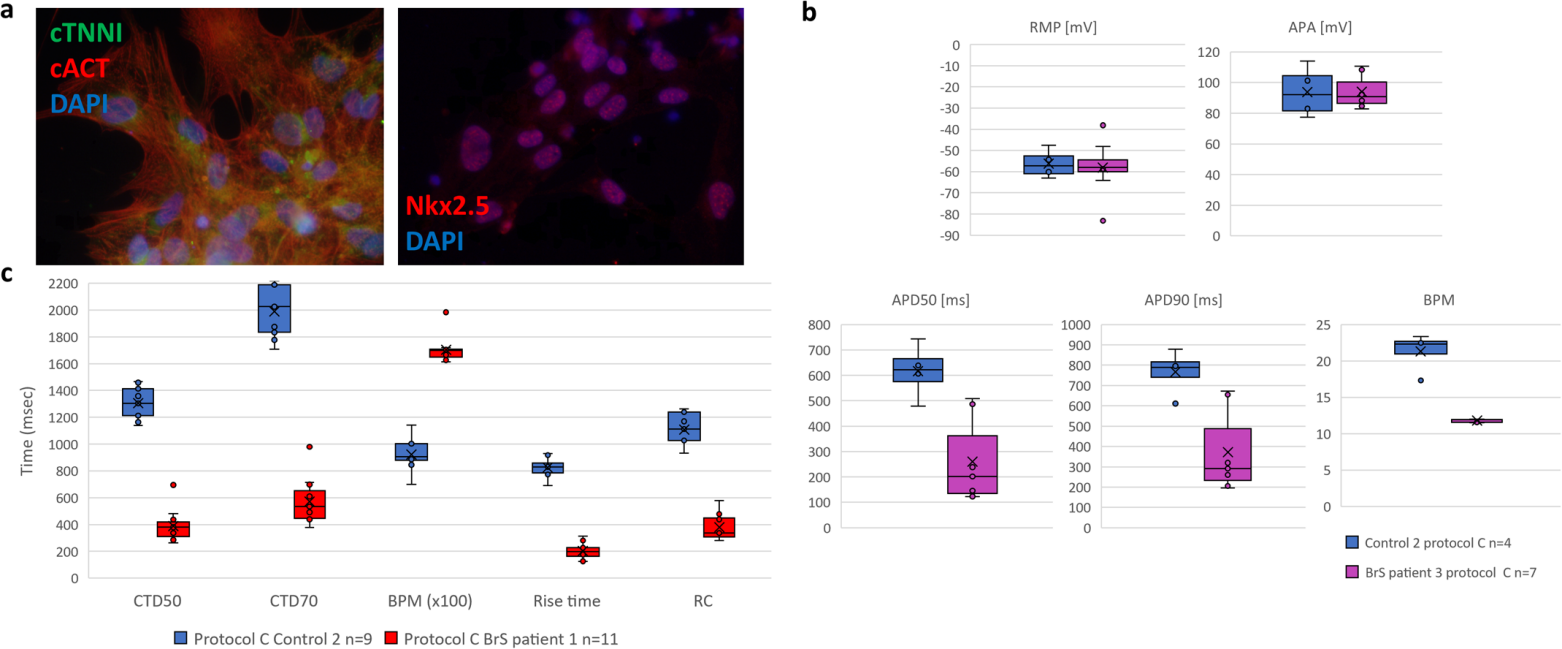
**Results obtained from iPSC-CM in the validation experiment of protocol B.** (A) Representative immunofluorescence staining of cardiac markers cTNNI, cACT and Nkx2.5 on non-dissociated iPSC-CMs; nuclei were visualized using DAPI. (B) AP properties of obtained iPSC-CMs. Displayed from left to right are box plots presenting the range of values for RMP, APA, APD90 and APD50 and BPM with indicated average (x), median (vertical line) and SEM (straight error bar) values for: Control 2 in blue (*n*=4) and BrS patient 2 in purple (*n*=7). (C) CT properties of obtained iPSC-CMs. Displayed from left to right are the box plots showing ranges of values for CTD50, CTD70, BPM, rise time and RC with indicated average (x), median (vertical line) and SEM (straight error bar) for Control 2 in blue (*n*=9) and BrS patient 1 in red (*n*=11).

Differentiation efficiency was calculated from cACT staining (Control 2: 99.7%; BrS patient 1: 74.7%; no ICC was performed for BrS patient 2 as due to obtaining contracting cells in only two out of six replicates those cells were used for AP recordings and RNA extraction; Table 2). We did observe expression of all cardiac markers (cACT, cTNNI and Nkx2.5) on protein level (Fig. 4A; unmerged images of cACT and cTNNI staining are presented in Fig. S4). Expression levels of six of the tested markers (*ANK3*, *HCN4*, *KCND3*, *KCNQ1*, *RYR2* and *TNNI3*) showed significant differences in comparison with the LV tissue (Fig. 3B; Table S3).

We recorded APs from two out of three differentiated iPSC-CM lines (Control 2 and BrS patient 2) (Fig. 4B; Table S5). For BrS patient 1 we mostly obtained contracting iPSC-CMs formed on sides of the coverslips, which were not accessible for patch-clamping experiments. iPSC-CMs from both cell lines showed similar RMP values (-56.3±3.4 and −60.9±4.1 for Control 2 and BrS patient 2, respectively; *P*=0.76) and amplitude (94±4 for BrS patient 2 and 93.9±8.4 mV for Control 2; *P*=0.992). We observed that APD was shortened for BrS patient 2 at both 90% and 50% of repolarization [twofold shortening for both APD50 (*P*=0.0023) and APD90 (*P*=0.025)] (Fig. 4B; Table S5).

In this validation experiment, we performed calcium imaging on the iPSC-CMs, an additional more high-throughput electrophysiological characterization. We collected CT recordings from Control 2 and BrS patient 1 (the contracting cells of BrS patient 2 were used for AP characterization and RNA extraction; Fig. 4C). Similar to our observations from AP recordings, CTD values for BrS patient 1 were about threefold shorter in comparison to those from Control 2 (*P*≤0.001) (exact values provided in Table S5). We could also see that BrS patient 1 showed shorter rise time and shorter transient decay in comparison to Control 2 [rise time: 202.3±17.9 ms versus 824.3±24.6 ms versus (*P*≤0.001); RC: 380.3±28.9 ms versus 1108.1±43.4 ms (*P*≤0.001), respectively] (Table S5).

Based on the obtained results we conclude that iPSC-CMs generated with selected protocol B are amenable for patch-clamping as well as more high-throughput calcium imaging characterization. Moreover, with the applied techniques we were able to detect differences in both APD and CTD between tested patient and control iPSC-CMs.

## DISCUSSION

Generation of appropriate iPSC-CMs is an important step towards cellular modelling of cardiac arrhythmias. Multiple monolayer-based differentiation protocols have been reported, but to date the comparative performance of such protocols in generating iPSC-CMs from several iPSC cell lines from different individuals was not tested. In this report we selected two protocols with addition of the same small molecule (CHIR99021) to induce mesoderm formation, whilst cardiac lineage differentiation was obtained with the use of different Wnt pathway inhibitors: IWP2 in protocol A and Wnt-C59 in protocol B, respectively (Fig. 1B). In addition, protocol A switched from supplementation of media with B27 supplement lacking insulin to B27 supplement with insulin on day 5 of differentiation, while in protocol B B27 supplement without insulin was used throughout the entire differentiation (Fig. 1C).

We generated iPSC lines from fibroblasts of two healthy donors as well as two BrS patients, and derived iPSC-CMs using the two selected protocols. In a comparison experiment, protocol A led to derivation of cells with a mixed patchy monolayer and 3D clumps morphology (Table 1). Cells generated with protocol B showed better organization of cTNNI on protein level (Fig. 2; Fig. S2).

In 2017, Jeziorowska et al. published a report in which they compared efficiencies of two monolayer-based differentiation protocols (implementing 6 μM CHIR99021 and 5 μM IWP2 or 2.5 μM IWR1, similar to our protocol A which involved addition of IWP2) with further maintenance in RPMI supplemented with B27 without insulin on three independent iPSC clones derived from a single control fibroblast line (Jeziorowska et al., 2017). They observed first contractions on day 8 and patchy monolayer morphology in iPSC-CMs obtained with both protocols. In our hands, first contractions occurred starting from day 7 in both protocols A (IWP2) and B (Wnt-C59), while the observed morphology was more protocol specific and varied from monolayer/patchy monolayer to 3D cell clumps (Fig. 1C and Table 1) but with the best monolayer formation in our protocol B.

We obtained contracting iPSC-CMs with comparable differentiation efficiency. Jeziorowska et. al. also observed that both tested monolayer-based protocols performed with similar differentiation efficiency (54±20% and 54±22% of cTNNI positive iPSC-CMs for IWP2 and IWR1, respectively) but lower than what we observed (≥90% of cardiac α-actinin positive iPSC-CMs; Table 1 and Table S2) (Jeziorowska et al., 2017). Nonetheless, in our hands, iPSC-CMs produced by protocol B were of better quality and more accessible for patch-clamp experiments. This was not only observed in the number of analyzed recordings with RMP <−50 mV, but as well in the total number of obtained AP recordings (Table S4). This could be explained by the presence of 3D structures formed in iPSC-CMs generated with protocol A, which hampered electrophysiological characterization, as in our experiment AP recordings were obtained from small undissociated clusters of cells (i.e. patches of monolayers). This way we mimicked better the physiological conditions, where cell–cell connections are intact and connecting proteins are structurally similar to that in cardiac tissue, as opposed to an isolated cell (Van de Sande et al., 2021). We checked if the obtained AP characteristics of iPSC-CMs from protocol B fall within the normal ranges, based on the AP properties described for iPSC-CMs in seven previous literature reports, where differentiation protocols and patch-clamping conditions similar to our report were used (Jeziorowska et al., 2017; Bett et al., 2013; Gibson et al., 2014; Meijer van Putten et al., 2015; Lopez-Redondo et al., 2016; Herron et al., 2016; Feaster et al., 2015). Either commercially available or in-house differentiated control iPSC-CMs were characterized (summarized in Table S6). In these seven studies the following ranges for the analyzed AP parameters were observed: RMP −75 to −41.6 mV; APA 64 to 121 mV; APD50 280 to 991 ms and APD90 135 to 1216 ms. We concluded that although published iPSC-CMs were characterized on slightly later time points (0–7 days later; Fig. 1C), all of the obtained AP parameters from our iPSC-CMs fell within the published AP parameter ranges (irrespective of the used differentiation protocol or disease status) (Fig. 4B; Tables S4–S5).

Although iPSC-CMs show expression of cardiac markers, their relative level differs from this observed in mature cardiomyocytes. Expression pattern of iPSC-CMs usually resembles the pattern observed in human fetal/embryonic cardiomyocytes (Zhou et al., 2017; Karakikes et al., 2015). We compared relative transcript expression of the selected cardiac markers in our iPSC-CMs with their expression in left ventricular tissue. Five of the markers: *KCND3*, *KCNQ1*, *RYR2*, *TNNI3* and *TNNT2* showed reduced relative expression in iPSC-CMs from both protocols (Fig. 3A; Table S3, Fig. S3). Similarly, Jeziorowska et al. reported about 70% reduced relative expression of *RYR2* in their iPSC-CMs derived using monolayer-based protocols. We observed reduced relative expression of *KCNH2*, *KCNJ8*, *MYH6* and *MYH7* and increased expression of *KCNJ2* and *GJA1* in iPSC-CMs generated with protocol A (Fig. 3; Fig. S3A, Table S3). Those observations suggest immaturity of the obtained iPSC-CMs, as those cardiac markers (*GJA1*, *KCND3*, *KCNH2*, *KCNJ8*, *KCNQ1*, *MYH7*, *RYR2*, *TNNI3*, *TNNT2*) have been reported to be expressed at higher levels in mature iPSC-CMs, obtained during long term culture or after hormonal maturation treatment (Zhao et al., 2018; Lewandowski et al., 2018; Sottas et al., 2018; Bedada et al., 2016; Garg et al., 2018). In iPSC-CMs obtained in our validation experiment, we detected increased relative expression of *HCN4*, in comparison with the reference tissue (Fig. 3B; Fig. S3B). High expression of *HCN4* is a known characteristic of iPSC-CMs, which explains their high spontaneous beating ability (automaticity) (Goversen et al., 2018). Although overall the expression pattern of cells generated with protocol B implies still incomplete maturity, it resembled that of adult left ventricle more closely in comparison with the tested protocol A.

To understand the underlying cause of the different performance of the tested similar differentiation approaches, we looked at the differences between the protocols. A different small molecule was used for cardiac fate determination, IWP2 in protocol A and Wnt-C59 in protocol B. The mechanism of action of both molecules is based on inhibition of a membrane-bound O-acyltransferase porcupine (PORCN) activity, to prevent Wnt ligand palmitoylation and its subsequent secretion (Chen et al., 2009). Although both molecules show different potency (IC_50_ of 27 nM for IWP2 and 74 pM for Wnt-C59), the concentrations of each molecule used to obtain the inhibitory effect on PORCN in the tested protocols account for this difference (5 µM of IWP2; 2 µM of Wnt-C59). Thus, the differences in performance between the protocols is most likely not attributable to the used small molecules. The remaining differences between the protocols included: (i) inclusion of a rest day before the cardiac fate determination (present in protocol A, absent in protocol B) and (ii) insulin switch (in protocol A) or absence (in protocol B). It was previously shown that insulin presence has an inhibitory effect on cardiomyocyte differentiation at early stages (between day 1–5 of differentiation) by its inhibitory effect on cardiac mesoderm formation, while its presence at later stages of differentiation promotes the oxidative metabolism, characteristic for mature cardiomyocytes (Lian et al., 2013b; Freund et al., 2008; Zhao et al., 2019). This is why in the tested differentiation protocol A, Lian et al. proposed an insulin switch on day 5 of differentiation (Lian et al., 2013a). Thus, the included rest day and introduction of insulin in the tested protocol A are the potential critical points in terms of the differences in the iPSC-CM morphology as well as molecular profile of the cells, in comparison to protocol B.

Finally, with results collected in an independent validation experiment we proved that protocol B generated iPSC-CMs with similar morphology and contracting cells from all of the tested cell lines, with efficiency ≥94% (Table 2). As calcium handling plays an important role in the electromechanical coupling of the human heart, we decided to implement calcium imaging in our experimental pipeline in the validation experiments. Intracellular calcium (Ca_i_^2+^) modulates Na^+^ current density without changing its kinetics in freshly isolated ventricular myocytes (Casini et al., 2009). Hwang et al. showed that iPSC-CMs derived from different iPSC lines reprogrammed using different approaches have similar calcium handling properties. Their CT properties are relatively mature around day 21 post differentiation and comparable to those of human native CMs (Hwang et al., 2015). We compared our CT parameters with those previously published in six independent reports (Shah et al., 2019, 2020; Liang et al., 2016) (summarized in Table S7). These six papers revealed the following ranges for CT parameters: CTD90 1093–2897.5 ms; CTD50 459–1633 ms; rise time 85–500 ms and transient decay 230–2937 ms. The CT values (CTD50, rise time and transient decay) obtained from our iPSC-CMs generated from both Control 2 and BrS patient 1 iPSC-CMs fitted within the range of previously published data (Fig. 4C; Table S5). It has previously been shown that calcium transient duration is usually longer than APD (1.2–2 times longer) (Prajapati et al., 2018) and this property was also noted in our electrophysiological data for Control 2 iPSC-CMs (Fig. 4C; Table S5). We noticed significant APD and CTD shortening for BrS patient lines in comparison to our Control 2 iPSC-CMs [twofold shorter APD50 (*P*=0.0023) and 3.4-fold shorter CTD50 (*P*≤0.001)] and we also observed a shorter rise time (*P*≤0.001) and slower transient decay (*P*≤0.001) in CT recordings of BrS patient 1 in comparison to Control 2 (Fig. 4B,C). Additional patch clamp, sodium current measurement and calcium imaging experiments on BrS patients with the identical *SCN5A* founder mutation are needed in the prospective experiments to confirm these observations.

In the methodology of differentiations used in this report we focused on comparison of basic differentiation conditions. As the expression profile of the tested cardiac markers of our iPSC-CMs suggests their immaturity, for future experiments additional maturation strategies will be applied on the cells, such as triiodothyronine and/or glucocorticoid hormone treatment, micropatterning, electrical and/or mechanical stimulation or addition of specific miRNAs (White et al., 2016; Yang et al., 2014; Ulmer et al., 2018; Nunes et al., 2013; Parikh et al., 2017).

In conclusion, we were able to generate functional iPSC-CMs using both selected protocols. Both protocols A and B show similar efficiency of differentiation and produce CMs with electrophysiological parameters falling within the range of previously reported values for control iPSC-CMs. Moreover, both protocols generate cells expressing all of the tested cardiac markers on protein and RNA level. For our prospective experiments, we decided to further optimize protocol B based on the findings that the obtained iPSC-CMs showed (i) more consistent cardiac markers expression between the differentiation batches, with a closer resemblance of that observed in the left ventricle, (ii) better sarcomeric protein organization, (iii) >50% survival after dissociation and (iv) monolayers that were more amenable for patch clamp and calcium transient experiments.

## MATERIALS AND METHODS

### Fibroblast reprogramming to iPSCs and validation

Commercial fibroblasts – BJ (ATCC^®^CRL-2522™) (referred to as Control 1) were thawed and cultured for 8 days prior to reprogramming. Skin biopsies from one healthy control donor (Control 2) and two BrS patients (BrS patient 1 and 2 –carrying the same *SCN5A* variant: a Belgian BrS founder mutation c.4813+3_4813+6dupGGGT; Sieliwonczyk et al., 2021) were obtained using standard procedures and processed on the day of collection as follows: after mechanical cutting, sample underwent enzymatic digestion in 37°C in a mixture of trypsin (Life Technologies) and collagenase type IV (Sigma-Aldrich) (1:1) for 1 h. Cells were brought to culture in a T25 flask (Corning) in fibroblast culture media consisting of RPMI1640 media (Life Technologies) supplemented with 10% FBS (Life Technologies), 1% of Penicillin/Streptomycin (Life Technologies), 1% of sodium pyruvate (Sigma-Aldrich) and 0.1% of primocin (InvivoGen Europe), and cultured in 5% CO_2_ humidified conditions in 37°C up to 3 weeks.

Before transduction, cells were tested for absence of Mycoplasma DNA (LookOut^®^ Mycoplasma PCR Detection Kit, Sigma-Aldrich) according to the supplier's protocol. Cells were cultured on tissue culture treated dishes (CORNING CoStar) in 37°C with 5% CO_2_. Reprogramming of the fibroblasts was performed using the CytoTune™-iPS 2.0 Sendai Reprogramming Kit and Essential 8 Flex media (Life Technologies), according to the protocol provided by the supplier for feeder-free cell culture. iPSC colonies were selected manually throughout ten passages [five passages using single colony picking and further on at least five passages were performed using Versene EDTA solution (Lonza) according to suppliers' protocol], from day 21 onwards on plates coated with extracellular matrix (Matrigel, CORNING) diluted in DMEM F12 media (Life Technologies) according to the suppliers' protocol (Fig. 1A) (Santostefano et al., 2015).

The obtained iPSCs were validated confirming the presence of pluripotency markers [Nanog (Cell Signaling Technology), Oct 3/4 (Cell Signaling Technology), Tra 1-60 (Life Technologies), Tra 1-81 (Life Technologies)] with immunofluorescence staining on non-dissociated iPSC colonies fixed on passage 10 (P10) (Fig. S1, Tables S8–S9), performing a qPCR analysis to prove absence of Sendai virus vectors and running SNP arrays to confirm retention of genomic integrity, using protocols described below. For long term preservation in liquid nitrogen, cells were frozen down in KnockOut Serum Replacement (Life Technologies) supplemented with 10% DMSO (Sigma-Aldrich).

### Immunofluorescence staining

iPSCs/iPSC-CMs for immunofluorescence staining were grown on glass coverslips (Saillart Glass Atelier). iPSCs on P10 or iPSC-CMs on D20-30 were fixed by incubation with 98% ice cold methanol (Sigma-Aldrich) for 20 min in −20°C and washed afterwards with PBS (Life Technologies). Permeabilization of the cells was carried out for 15 min with PBS supplemented with 0.1% Triton X-100 (Sigma-Aldrich). Subsequently cells were incubated with the blocking solution at room temperature for 30 min for iPSCs and 1 h for iPSC-CMs. Primary antibodies were diluted in DAKO real diluent (DAKO) and cells were incubated overnight at 4°C. Secondary antibodies were diluted in DAKO Real antibody diluent and cells were incubated for 1 h to stain pluripotency markers [Nanog (Life Technologies), Oct 3/4 (Santa Cruz Biotechnology), Tra 1-60 (Cell Signaling Technology), Tra 1-81 (Cell Signaling Technology)] or cardiac specific markers [cardiac α-actinin (cACT) (Abcam), cardiac troponin I (cTNNI) (Abcam) and Nkx2.5 (Abcam)] at room temperature. The composition of blocking solutions and dilutions of each antibody used are listed in detail in Table S8 for pluripotency and Table S9 for cardiomyocyte markers. Nuclei were visualized by 5 min incubation with DAPI (Life Technologies) in a 1 in 1000 dilution in PBS. Before imaging, cells were mounted with mounting solution Fluoromount-G (Life Technologies). Fluorescent images were acquired at room temperature using an Olympus BX51 fluorescence microscope equipped with an Olympus DP71 digital camera. cellSens software was used for image acquisition and processing.

### Sendai vectors presence test

Prior to the differentiation experiments, selected clones were validated for the absence of the reprogramming vectors. The cell pellets containing 5×10^5^-1×10^6^ cells were collected on day 0 of reprogramming (Sendai negative control), day 7 of reprogramming (Sendai positive control) and passage 10 during reprogramming from each of the validated clones for each reprogrammed cell line. Pellets were resuspended in RNA lysis buffer and frozen down in −80°C prior to extraction. RNA was extracted using RNA extraction kit (Zymo) and cDNA was prepared using Super Script III First Strand Synthesis kit (Life Technologies) according to the manufacturer's protocols.

Sendai virus presence PCR was carried out using primers from the sequences provided by the supplier in User Guide: Cyto Tune™-iPS 2.0 Sendai Reprogramming Kit using Taq Polymerase and Buffer (Life Technologies) using the PCR protocol described in Table S10. The iPSC clone was considered Sendai negative when primers for Sendai virus backbone and at least two out of three other primer pairs showed a negative result.

### Genomic integrity test using SNP array

DNA sample was collected from: fibroblasts on D0 of reprogramming and iPSC clones on passage 10 before differentiation to CMs. DNA was extracted using an automatic DNA extraction system Maxwell^®^ RSC with Maxwell^®^ RSC Cultured Cells DNA Kit (Promega), following the supplier's protocol. Briefly, 5×10^5^–1×10^6^ cells were collected from cell culture dishes and centrifuged at 120 rcf for 5 min at room temperature. The supernatant was discarded, and cell pellet was frozen down in −20°C until the day of extraction. Cell pellet was thawed at room temperature and resuspended in the media residues present in the sample. One Maxwell^®^ RSC Cultured Cells DNA Cartridge was used per sample. First well of the DNA Cartridge was loaded with the cell sample and mixed by pipetting with the lysis buffer in the well. Elution tubes on the deck tray were filled with 100 μl of Elution Buffer. Extraction was carried out using Maxwell RSC Instrument using the Cultured Cells DNA method and DNA sample was stored at +4°C after extraction.

HumanCytoSNP-12 array (Illumina) was run according to the supplier's protocol for the automated Infinium HD Assay Ultra. Genome Studio (Illumina) software was used to visualize results and confirm identity between the iPSC clones and original fibroblast line. Results were further analyzed with CNV-WebStore, an in-house developed online available CNV Analysis tool (http://cnv-webstore.ua.ac.be). Large novel CNVs (>100 kb) detected in the used iPSC clones, which were absent in the original fibroblast cell lines are presented in Table S1.

### Cardiomyocyte differentiation

Prior to differentiation, cells underwent two to three expansion passages using Versene/EDTA 0.02% solution (Lonza) and final passage on six-well cell culture treated plates (CORNING CoStar) with glass coverslips (Saillart, Glass Atelier), coated with Matrigel (Corning). iPSCs were cultured in an incubator with humidified conditions at 37°C and 5% CO_2_ until they reached 80–90% confluency before starting the differentiation procedure. Both differentiation protocols started on day 0 with RPMI1640 (Life Technologies) media supplemented with 2% B27 supplement without insulin (Life Technologies) and CHIR99021 (6 µM, Axon Medchem) to induce mesodermal differentiation (Fig. 1B,C). The next steps for each protocol were as follows:

Protocol A – previously reported by Lian et al. (2013a), briefly: on day 3 of differentiation, RPMI1640 medium with 2% B27 supplement without insulin and IWP2 (5 μM, SelleckChem) was added to the cells for 48 h. Cells were subsequently cultured in RPMI1640 medium with 2% B27 supplement with insulin (Fig. 1C).

Protocol B – previously described by Burridge et al. (Burridge et al., 2014), briefly: on day 2 of differentiation cells were cultured in RPMI1640 medium with 2% B27 supplement without insulin and Wnt-C59 (2 μM, SelleckChem) for the next 48 h. Cells were subsequently cultured in RPMI1640 medium with 2% B27 supplement without insulin (Fig. 1C).

From day 0 of differentiation, cells were cultured in an incubator with humidified atmosphere at 37°C and 5% CO_2_. Cells were cultured until D20 of differentiation before performing further experiments. For RNA collection and single-cell patch-clamp recordings, dissociation of iPSC-CMs was performed using Accumax or TrypLE (Life Technologies) according to the suppliers' protocol.

Undissociated cells were fixed on D20-25 of differentiation and immunofluorescence staining for cardiac specific markers [cardiac α-actinin (cACT), cardiac troponin I (cTNNI) and Nkx2.5] was performed according to the protocol described in detail in the Immunofluorescence staining section of the Materials and Methods. Analysis of iPSC-CMs quality was performed based on the expression of cardiac specific markers on RNA level, as well as expression and organization of the sarcomeric proteins based on immunofluorescence staining. Electrophysiological properties were characterized from AP/CT recordings to investigate signal propagation in iPSC-CMs and their general electrophysiological activity.

### Electrophysiological experiments

Perforated-patch AP recordings were performed 25–28 days after the start of differentiation at room temperature (20–22°C) on non-dissociated cells (unless stated otherwise) grown on glass coverslips using the current clamp mode of the Axopatch 200B amplifier and a pClamp 10.7/Digidata 1440A acquisition system (Axon Molecular Devices). Patch-pipettes with a resistance of approximately 2 MΩ were pulled from 1.2 mm borosilicate glass capillaries (World Precision Instruments, Inc.) using a P-2000 puller (Sutter Instrument Co.). Pipettes were filled with an intracellular solution containing (in mM): 4 NaCl, 106 KCl, 5 K_2_ATP, 2 MgCl_2_, 5 K_4_BAPTA and 10 HEPES adjusted to pH 7.2 with KOH and addition of 0.72 ng/ml amphotericin B (A9528, Sigma-Aldrich). The cells were continuously superfused at 1 ml/min rate with an extracellular solution (ECS) containing (in mM): 150 NaCl, 5.4 KCl, 1.8 CaCl_2_, 1 MgCl_2_, 15 HEPES, 15 glucose, 1 Na-pyruvate adjusted to pH 7.4 with NaOH. Junction potentials were compensated prior to sealing of the cell before the patch pipette made contact with the cell membrane.

AP waveforms were recorded using the current clamp mode under the whole-cell perforated patch configuration without pacing. Spontaneous APs were recorded over 10 s per sweep. Recordings were carried out for maximum 10 min after obtaining perforated cell access.

Intracellular Ca^2+^ transients (CT) were registered from cell populations on an epifluorescent microscope setup using a Photomax 200 detection system (DAGAN). The iPSC-CMs were loaded with 5 µM Cal-520 AM (Abcam, ab171868) fluorogenic calcium sensitive dye and 1% Pluronic™ F-127 (20% solution in DMSO, Life Technologies, P300MP) supplemented to the culture medium. After incubation for 60–90 min (37°C, 5% CO_2_), the cells were washed with pre-warmed ECS and incubated for 30 min to allow recovery before imaging. Experiments were performed on non-dissociated cells at D25-28 on glass coverslips using the same ECS solution as for AP recordings.

Electrophysiological data was analyzed using PClamp10 software (Axon CNS Molecular devices). For each cell recording, AP and CT values were calculated as a mean of five subsequent waveforms and data are presented as a mean±s.e.m. of the means for each single cell of the ‘n’ cells analyzed. The following parameters were analyzed: APA, RMP, APD50 and APD90. The time between two AP peaks was presented as BPM (60/time in seconds). APD correction related to beating rate was not performed, as the obtained iPSC-CMs were all beating slower than 60 BPM. The amplitudes of Ca^2+^ transients are presented as a pseudo difference ΔF=F - F_base_ where the F and F_base_ are the measured fluorescence intensity at the peak and before/after the CT. To determine the rate constant of decay (RC), the slope after the ΔF_max_ to F_base_ was fitted using standard exponential function: 
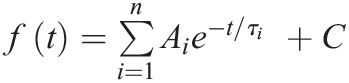
. Comparisons between groups were performed using one-way ANNOVA (Systat Software Inc.). A *P*-value of ≤0.05 is considered statistically significant.

### qPCR

RNA was extracted from iPSC-CMs on D20-25 of differentiation, using RNA Extraction Kit (Zymo) and cDNA was prepared using Super Script III First Strand Synthesis kit (Life Technologies) according to the manufacturer's protocols. Quantitative RT-PCR of cardiac markers: *ANK2*, *ANK3*, *CACNA1C*, *GJA1* (*CNX43*), *HCN4*, *KCND3*, *KCNH2*, *KCNJ2*, *KCNJ8*, *KCNQ1*, *MYH6*, *MYH7*, *RYR2*, *TNNT2*, *TNNI3* was performed using in-house designed primers listed in Table S11 and qPCR MasterMix Plus for SYBR Assay No-ROX (Tebu-bio N.V.). Each reaction was performed in triplicate and fold changes in gene expression were determined using the comparative CT method (ΔΔCt) with normalization to three reference genes: *GAPDH*, *ECHS1* and *RPL13A* and relative to the expression in iPSCs. Results were analysed using QBase 3.2 (Biogazelle). As ventricular cardiomyocytes are the cell type of interest to study inherited arrhythmia disorders, healthy human heart donor left ventricle (LV) tissue expression is shown as a reference in relative expression graphs and the expression profile of iPSC-CMs generated with each protocol was statistically compared with the reference tissue.

Comparison of the normalized Ct values between the groups (protocols and reference tissue) was performed with a linear mixed model, having the protocol as fixed effect and the normalized Ct value as dependent variable. The nonindependence between observations within the same cell line was accounted for using a random intercept for cell line, and a random slope for protocol. Pairwise comparisons between the protocols and/or reference tissue were carried out using a post hoc analysis with Tukey's correction for multiple hypothesis testing. Analyses were carried out in the software package R version 4.0.2, and the add on packages Ime4 and multcomp. A *P*-value of ≤0.05 is considered statistically significant.

### Imaging data analysis

The light microscopy images of contracting iPSC-CMs were acquired at a rate of 20 frames per second using cellSens software (Olympus LS) and converted to TIFF images for analysis using Fiji image analysis freeware (Schindelin et al., 2012), with manual indication of the areas of contracting cell monolayer, based on the movements of the pixels in the generated stacks of images. The calculated contracting area sizes were measured in mm^2^ and divided by the area of the whole image of the culture surface (approximately 96 mm^2^).

Differentiation efficiency was calculated based on in total four randomly taken immunofluorescence staining images for cACT for each tested iPSC-CM line. Fluorescence images were analyzed using CellBlocks, a Fiji script for cell-based analysis (De Vos et al., 2010). In brief, individual nuclei were detected using a Laplacian operator and automatic thresholding on the DAPI-channel. After particle analysis and dilation of the nuclear objects, the resulting regions of interest were used to measure the signal intensities in the cACT on the GFP channels within 50 µM around the nuclei. User-defined thresholds were set to classify cells as being positive for either marker, calibrated to commercial hSC-CMs (Van de Sande et al., 2019) as a positive control and negative fibroblast cACT expression. Efficiency is presented as percentage of cells positive for cACT.

## Supplementary Material

Supplementary information
