# Supplementary material for: Morpho-functional comparison of differentiation protocols to create iPSC-derived cardiomyocytes
Source: Biol Open. 2022 Feb 23;11(2):bio059016. doi: 10.1242/bio.059016 (PMC8890088; doi:10.1242/bio.059016)
Supplement: Supplementary information [file biolopen-11-059016-s1.pdf]

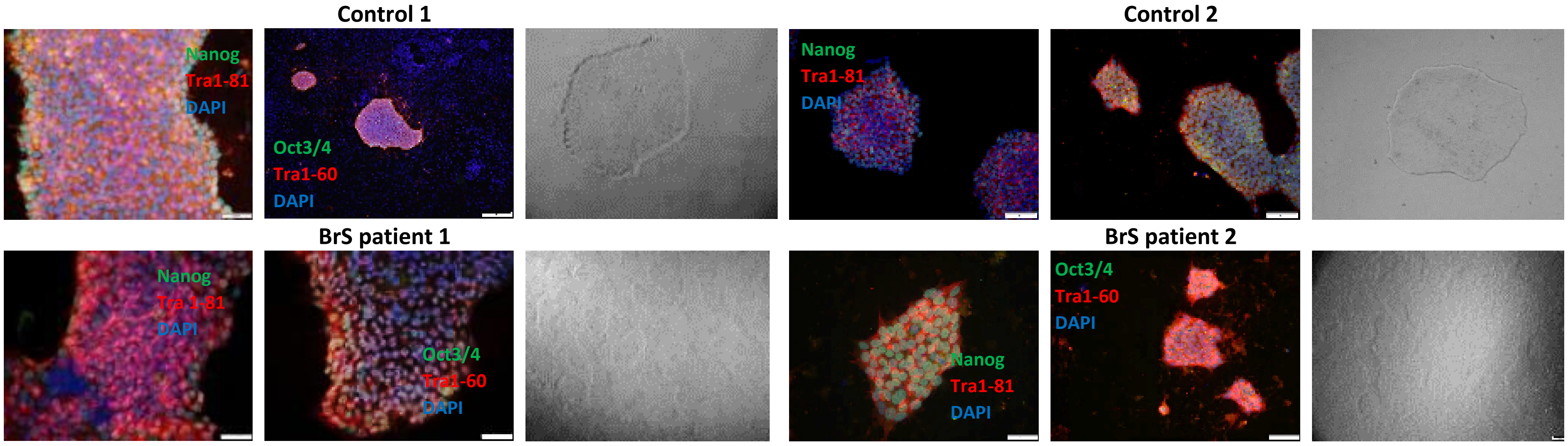

Fig. S1. Bright field images and representative immunofluorescence staining of pluripotency markers of iPSCs of Control 1, Control 2, BrS patient 1 and BrS patient 2.

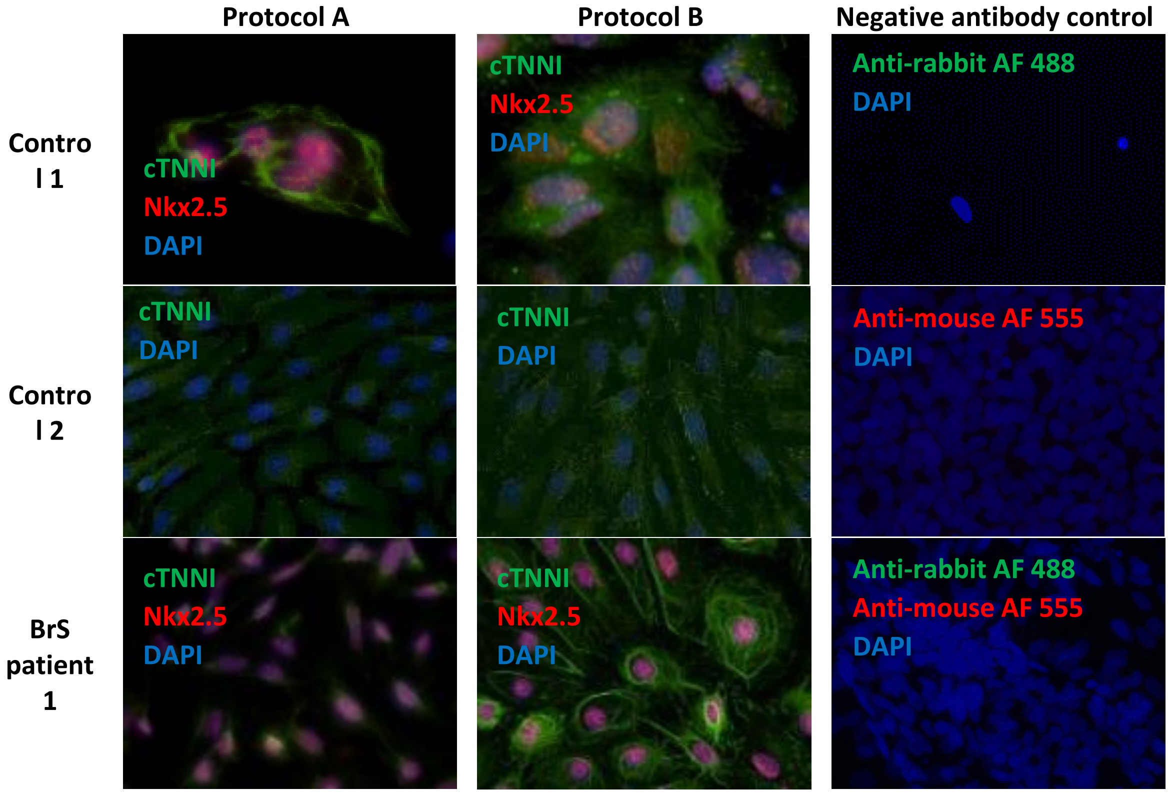

Fig. S2. Immunofluorescence staining of cTNNI of iPSC-CMs of Control 1, 2 and BrS patient 1 generated with protocol A and B in the comparison experiment. No cTNNI or Nkx2.5 staining was performed on iPSC-CMs from BrS patient 2 due to low amount of contracting areas on coverslips, which were used for electrophysiological tests and ICC staining for cACT. Negative secondary antibody controls (anti-rabbit Alexa Fluor 488 and/or anti-mouse Alexa Fluor 555) are shown in the right column.

Biology Open • Supplementary information

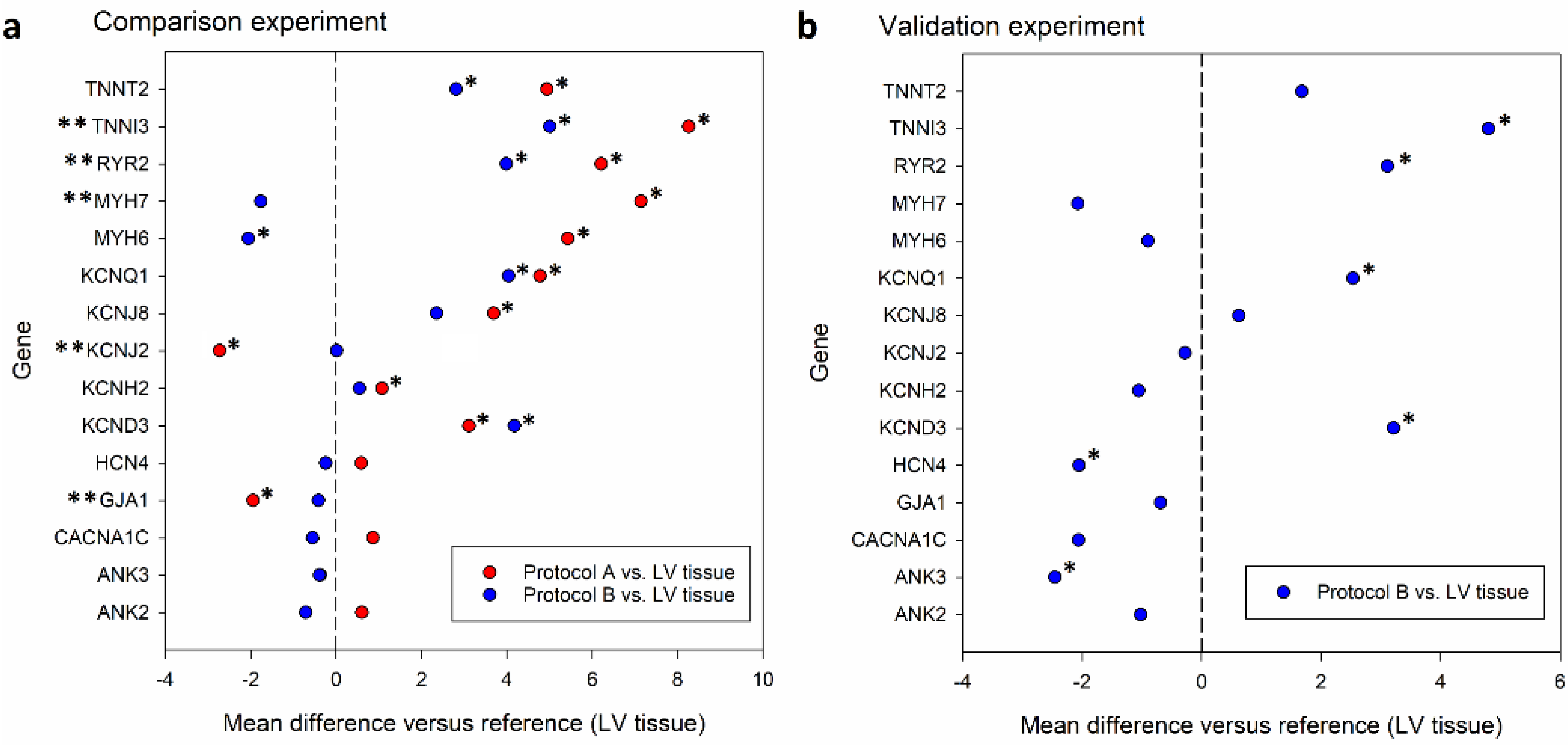

Fig. S3. Measured Ct values for all tested genes in iPSC-CMs generated using protocol A (in red) and protocol B (in blue) in relation to left ventricle (0 level on the x axis) from samples obtained in the comparison (a) and validation (b) experiment.
The figures show graphical overview of the difference in the measured mean Ct values. Values for iPSC-CMs generated with protocol B in comparison experiment show closer resemblance to those in the reference tissue in comparison to iPSC-CMs generated with protocol A. *p≤0.05 for Ct comparison between the used protocol versus reference tissue (left ventricle of the healthy donor heart); **p≤0.05 for Ct comparison between protocols A and B.

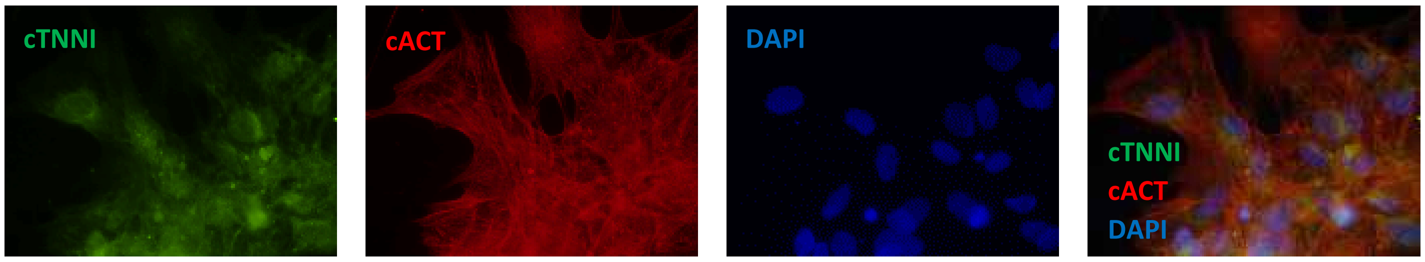

Fig. S4. Unmerged immunofluorescence staining images of cardiac markers cACT and cTNNI obtained from iPSC-CMs in the validation experiment (merged images are presented in Fugure 4a).

Biology Open • Supplementary information

Table S1. CNVs (larger than 100kb) detected in the used iPSC-lines.

Cell line	CNV type	CNV minimal region	CNV size (bp)	Genes in the region
Control 1	del	Chr1:1,030,565-1,266,041	235477	ACAP3, mir429, TTLL10
	del	Chr1:2,532,899-2,745,883	212985	MMEL1
	del	Chr1:191,618,129-191,875,752	257624	
	del	Chr4:994,414-1,188,049	193636	FGFRL1
	del	Chr4:34,049,908-34,423,079	373172	LINC02484
	del	Chr4:43,363,694-43,526,808	163115	LINC02383
	del	Chr4:113,294,328-113,779,891	485564	ALPK1, mir367
	del	Chr6:27,002,406-27,194,072	191667	HIST1H2AG
	del	Chr8:78,359,134-78,510,649	151516	LOC102724874
	del	Chr8:144,669,392-144,861,985	192594	BREA2, PYCR3
	del	Chr8:144,998,779-145,268,000	269222	CYC1, MROH1
	dup	Chr10:54,002,021-54,141,652	139632	DKK1
	del	Chr11:39,066,761-39,266,095	199335	
	del	Chr14:105,285,159-105,395,204	110046	CEP170B
	del	Chr19:1,341,509-1,497,060	155552	APC2
	del	Chr19:1,566,043-1,712,898	146856	MBD3
	dup	ChrX:71,284,941-71,411,659	126719	FLI44635
	dup	ChrX:91,685,124-92,178,902	493779	PCDH11X
Control 2	del	Chr16:32,624,879-32,855,389	230511	TP53TG3
	dup	Chr19:54,737,010-54,841,732	104723	LILRA3
	dup	ChrX:48,645,256-48,746,726	101471	ERAS
	dup	ChrX:153,051,907-153,197,130	145224	ARHGAP4
BrS patient 1	-	-	-	-
BrS patient 2	dup	Chr9:2,407,101-2,618,969	211869	VLDLR-AS1
	del	Chr10:68,196,544-68,517,908	321365	CTNNA3
	del	Chr11:90,802,950-90,926,931	123982	
	del	Chr16:32,564,812-32,959,259	394448	SLC6A10P
	del	ChrX:7,564,428-7,736,547	172120	

Del = deletion, dup = duplication

Biology Open • Supplementary information

Table S2. Differentiation efficiency based on immunofluorescence staining for cACT (comparison experiment).

	Protocol A		Efficiency	Protocol B		Efficiency
	Dissociated iPSC-CMs			Dissociated iPSC-CMs		
**Control 1**	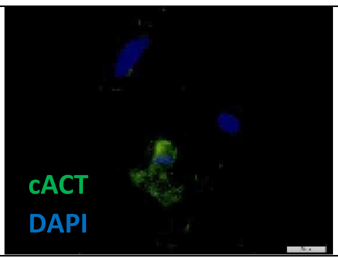		**80%**	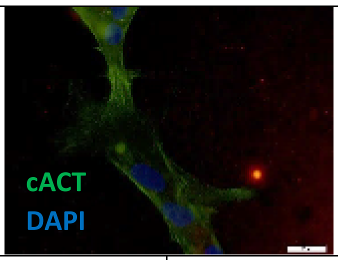		**100%**
	Non-dissociated monolayer			Non-dissociated monolayer	Non-dissociated monolayer	
**Control 2**	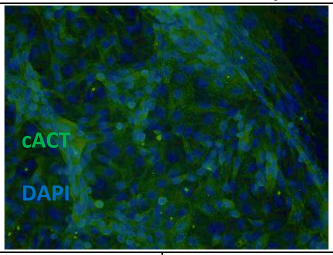		89.5 %	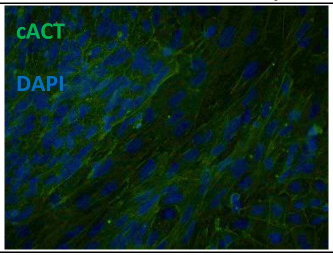	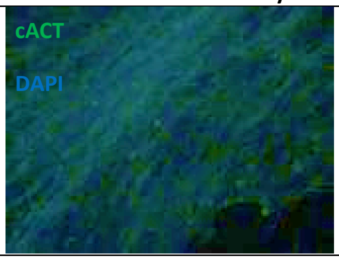	83.65 %
	Non-dissociated monolayer	Dissociated iPSC-CMs		Non-dissociated monolayer	Dissociated iPSC-CMs	
**BrS patient 1**	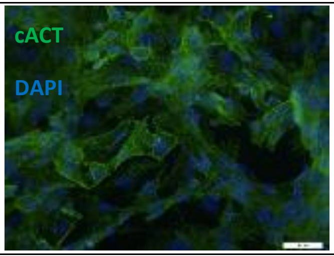	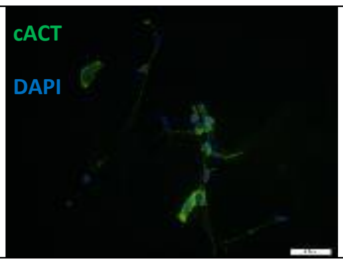	91.2 %	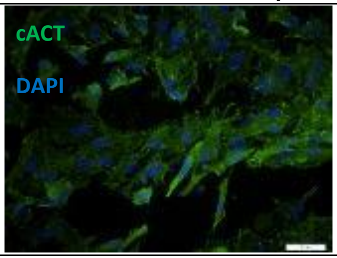	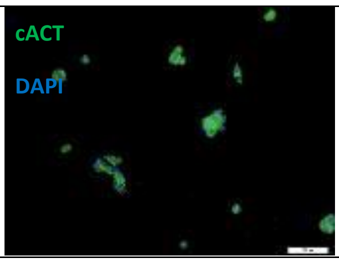	100 %
	Dissociated iPSC-CMs			Dissociated iPSC-CMs		
**BrS patient 2**	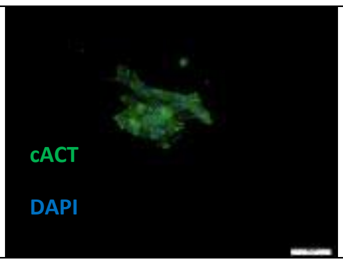		100 %	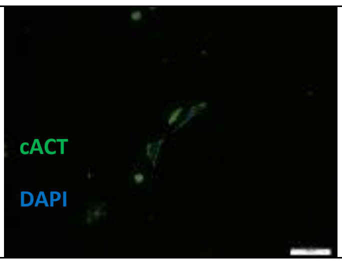		100 %

Biology Open • Supplementary information

Table S3. Calculated p values for the tested cardiac markers for qPCR data on samples from the comparison between the two protocols and in relation to the reference tissue (left ventricle of a healthy human heart donor).

Tested gene	Protocol A vs. Protocol B	Protocol A/B vs. reference tissue		
	Comparison round	Comparison experiment		Validation experiment
		Protocol A	Protocol B	Protocol B
ANK2	0.155	0.059	0.414	0.323
ANK3	0.985	0.505	0.652	1.7*E^{-6}*
CACNA1C	0.349	0.087	0.699	0.115
GJA1	7.5*E^{-5}*	3.6*E^{-5}*	0.239	0.6
HCN4	0.453	0.439	0.776	4.6*E^{-6}*
KCND3	0.057	1*E^{-6}*`	2.2*E^{-6}*	1.1*E^{-9}*
KCNH2	0.570	0.010*	0.539	0.176
KCNJ2	7.8*E^{-4}*	8.9*E^{-5}*	0.983	0.413
KCNJ8	0.288	1.1*E^{-16}*	0.081	0.504
KCNQ1	0.491	5.2*E^{-6}*	0.002*	0.027*
MYH6	0.105	0.016*	0.013*	0.285
MYH7	0.015*	0.002*	0.171	0.539
RYR2	0.042*	2. 8*E^{-6}*	9.9*E^{-4}*	4.87*E^{-5}*
TNNI3	0.026*	5.6*E^{-7}*	0.0023*	0.035*
TNNT2	0.138	1.7*E^{-4}*	0.037*	0.175

P-values were corrected for multiple testing using Tukey’s correction. *p≤0.05

Table S4. Action potential properties for iPSC-CMs generated in the comparison experiment.

**Used protocol**	**Protocol A**			**Protocol B**		
**Cell line**	**Control 2**	**BrS patient 1**	**BrS patient 2**	**Control 2**	**BrS patient 1**	**BrS patient 2**
**Number of AP recordings**	4	8	8	10	9	13
**Number of analysed AP recordings**	0*	0*/†	1	0*/‡	4	2
**APD50 [ms]**	-	-	422.08	-	351.51± 24.84	273.7± 40.83
**APD90 [ms]**	-	-	501.8	-	509.95± 27.91	422.3± 10.9
**APA [mV]**	-	-	114.5	-	90.8± 5.1	86.7± 17.3
**RMP [mV]**	-	-	-59.3	-	-59.6± 3.1	-58.3± 5.4
**BPM**	-	-	8	-	17± 1.12	27± 3

*Recordings with RMP >-40 mV; †Recordings with proper RMP but cells did not fire AP; ‡Declined seal quality due to vivid beating

**No proper recordings were obtained from Control 1 for both tested protocols.

Biology Open • Supplementary information

Table S5. Action potential and calcium transient properties for iPSC-CMs generated in validation experiment.

	Protocol B		
	Control 2	BrS patient 1	BrS patient 2
**Number of analysed AP recordings**	4	0	7
**APD50 [ms]**	617.2± 54.5	-	260.7± 59
**APD90 [ms]**	767.6± 56.4	-	371.5± 72.3
**APA [mV]**	93.9± 8.4	-	94± 4
**RMP [mV]**	-56.3± 3.4	-	-60.9± 4.1
**BPM**	21± 1	-	11± 0.3
**Number of analysed CT recordings**	9	11	0
**CTD50 [ms]**	1305.3± 41.9	389.7± 36.5	-
**CTD70 [ms]**	1988.9± 65.6	571.7± 52.1	-
**BD [s]**	6.62± 0.31	3.53± 0.06	-
**BPM**	9± 0.4	17± 0.3	-
**Rise time [ms]**	824.3± 24.6	202.3± 17.9	-
**RC [ms]**	1108.1± 43.4	380.3± 28.9	-

Table S6. Previously reported AP properties from commercial iPSC-CMs and iPSC-CMs differentiated using monolayer-based protocols

Reference	Differentiation protocol	Culture duration	Patching temperature	AP parameters				
				RMP	APA	APD50	APD90	n
Bett et. al. (2013)[1]	Commercial iCell CMs		RT	-63±5.8	83±11		955±103	25
Gibson et.al. (2014) [2]	Commercial iCell CMs		RT	-68±9	103±9		427±65	8
Meijer Van Puten et. al. (2015) [3]	Monolayer-based with CHIR 99021 and IWP4	30 days	36±1	-56±4	73±9		162±27	9
Lopez-Redondo et. al. (2016) [4]	Commercial iCell CMs		36±1	-43.3±1.7	79.4±2.9	309.4±29.5		54
Jeziorowska et. al. (2016) [5]	Monolayer-based using CHIR99021 and IWP2/IWR1	28-35 days	37	-69.5±9.5	96.6±15.5	586.8±404.3	726±489.6	156
Herron et. al. (2016) [6]	Commercial iCell CMs		37	-59.3±1.7	105±5			6
Feaster et. al. (2016) [7]	Monolayer-based with CHIR99021 and IWR1 + B27	30-35 days	RT	-74±1	118±3	326±13	378±14	53

Biology Open • Supplementary information

Table S7. Previously reported CT properties from iPSC-CMs differentiated using monolayer-based protocols.

Reference	Differentiation protocol	Culture duration	Imaging temperature	CT parameters				
				CTD90 [ms]	CTD50 [ms]	Rise time [ms]	Decay [ms]	n
Paci et. al. (2020) [8]	Commercial iCell^2 CMs		21±1	1826.2±1071.3	920.1±456.3			42
Shah et. al. (2020) [9]	END-2 co-culture	30 days	36±1	1900±100		480±20	250±20	68
Shah et. al. (2019) [10]	END-2 co-culture	30 days	36±1			251±29	2261±676	34
Higgins et. al. (2019) [11]	Monolayer-based with CHIR99021 and IWP2	60 days	36±1		1533±100		2790±50	200
Ahola et. al. (2017) [12]	END-2 co-culture	Not specified	36±1	1663±570	797±227			13
Liang et. al. (2016) [13]	Monolayer-based with CHIR99021 and IWR1	40-60 days	36±1		471.3±12.6	92±6.6		10-25

Table S8. Immunofluorescence blocking buffers and dilutions of used antibodies for pluripotency markers

**Blocking buffer**	PBS (Life Technologies)	Goat serum (Jackson Immunotechnologies; 5%)		
**Primary antibodies**	Nanog (PA1-097, Life Technologies; dilution 1:500)	Tra1-81 (4745S, Cell Signaling Technology dilution 1:200)	Oct3/4 (sc-9081, SantaCruz Biotechnology dilution 1:100)	Tra1-60 (4746S, Cell Signaling Technology dilution 1:200)
**Matching secondary antibody**	Goat anti-rabbit Alexa Fluor 488 (A-11034, Life Technologies; dilution 1:500)	Goat anti-mouse (IgM) Alexa Fluor 555 (A-21426, Life Technologies; dilution 1:500)	Goat anti-rabbit Alexa Fluor 488 (A-11034, Life Technologies; dilution 1:500)	Goat anti-mouse (IgM) Alexa Fluor 555 (A-21426, Life Technologies; dilution 1:500)

Table S9. Immunofluorescence blocking buffer and dilutions of used antibodies for cardiac markers.

**Blocking buffer CM markers**	Goat serum (Jackson Immunotechnologies; 5%)	PBS (Life Technologies)	
**Primary antibodies**	Cardiac α-actinin (Abcam; dilution 1:300)	Cardiac troponin I (Abcam; dilution 1:100)	Nkx2.5 (Abcam; dilution 1:200)
**Matching secondary antibodies**	Goat anti rabbit (Life Technologies; dilution 1:500)	Goat anti rabbit (Life Technologies; dilution 1:500)	Goat anti mouse IgG (Life Technologies; dilution 1:500)

Table S10. Sendai vectors presence test - PCR reaction protocol

**Initialization**	94°C	5 min	
**Denaturation**	94°C	15 sec	34x
**Annealing**	60°C	30 sec	
**Elongation**	72°C	45 sec	
**Final elongation**	72°C	10 min	
**Final hold**	10°C	1 min	

Table S11. Cardiac markers qPCR primers

	FORWARD 5'-3'	REVERSE 5'-3'
**REFERENCE GENES**		
**GAPDH**	TGCACCACCAACTGCTTAGC	GGCATGGACTGTGGTCATGAG
**ECHS1**	AAGGCCCTCAATGCACTTTG	ACTCAGGTTCTGCATTTCCTTG
**RPL13A**	CCTGGAGGAGAAGAGGAAAGAGA	TTGAGGACCTCTGTGTATTTGTCAA
**TARGET GENES**		
**ANK2**	TGGACTTCACAGCCAGGAAT	GCCTCGATCCAGTAAGAGCT
**ANK3**	ACCAAAGGAGGACAGCAACT	GAAAAGACAGACGACCACAGG
**CACNA1C**	TGACATCGAGGGAGAAAACT	ACATTAGACTTGACTGCGGC
**GJA1 (CX43)**	GGTGACTGGAGCGCCTTAG	GCGCACATGAGAGATTGGGA
**HCN4**	ACCCATGCTACAGGACTTCC	GAAGAGCGCGTAGGAGTACT
**KCND3**	AAACAATCACAGGGACTGGC	ACACCATTGTCACCATGACC
**KCNH2**	TCCTTCTCCATCACCACCTC	AAATCGCCTTCTACCGGAAA
**KCNJ2**	GTGCGAACCAACCGCTACA	CCAGCGAATGTCCACACAC
**KCNJ8**	AGTGGAATGGAGAAAAGTGGT	TCCTCTGTCATCATCCTCCC
**KCNQ1**	ACAAAGTACTGCATGCGTCG	CATGAGAACCAACAGCTTCG
**MYH6**	GATAGAGAGACTCCTGCGGC	CCGTCTTCCCATTCTCGGTT
**MYH7**	TCGTGCCTGATGACAAACAGGAGT	ATACTCGGTCTCGGCAGTGACTTT
**RYR2**	CATCGAACACTCCTCTACGGA	GGACACGCTAACTAAGATGAGGT
**TNNI3**	TGTGGACAAGGTGGATGAAG	CCGCTTAAACTTGCCTCGAA
**TNNT2**	GGAGGAGCTCGTTTCTCTCA	CCTCCTGTTCTCCTCCTCCT

Biology Open • Supplementary information

[1] G.C. Bett, A.D. Kaplan, A. Lis, T.R. Cimato, E.S. Tzanakakis, Q. Zhou, M.J. Morales, and R.L. Rasmusson, Electronic "expression" of the inward rectifier in cardiocytes derived from human-induced pluripotent stem cells. Heart Rhythm 10 (2013) 1903-10.
[2] J.K. Gibson, Y. Yue, J. Bronson, C. Palmer, and R. Numann, Human stem cell-derived cardiomyocytes detect drug-mediated changes in action potentials and ion currents. J Pharmacol Toxicol Methods 70 (2014) 255-67.
[3] R.M. Meijer van Putten, I. Mengarelli, K. Guan, J.G. Zegers, A.C. van Ginneken, A.O. Verkerk, and R. Wilders, Ion channelopathies in human induced pluripotent stem cell derived cardiomyocytes: a dynamic clamp study with virtual IK1. Front Physiol 6 (2015) 7.
[4] F. Lopez-Redondo, J. Kurokawa, F. Nomura, T. Kaneko, T. Hamada, T. Furukawa, and K. Yasuda, A distribution analysis of action potential parameters obtained from patch-clamped human stem cell-derived cardiomyocytes. J Pharmacol Sci 131 (2016) 141-5.
[5] D. Jeziorowska, V. Fontaine, C. Jouve, E. Villard, S. Dussaud, D. Akbar, V. Letang, P. Cervello, J.M. Itier, M.P. Pruniaux, and J.S. Hulot, Differential Sarcomere and Electrophysiological Maturation of Human iPSC-Derived Cardiac Myocytes in Monolayer vs. Aggregation-Based Differentiation Protocols. Int J Mol Sci 18 (2017).
[6] T.J. Herron, A.M. Rocha, K.F. Campbell, D. Ponce-Balbuena, B.C. Willis, G. Guerrero-Serna, Q. Liu, M. Klos, H. Musa, M. Zarzoso, A. Bizy, J. Furness, J. Anumonwo, S. Mironov, and J. Jalife, Extracellular Matrix-Mediated Maturation of Human Pluripotent Stem Cell-Derived Cardiac Monolayer Structure and Electrophysiological Function. Circ Arrhythm Electrophysiol 9 (2016) e003638.
[7] T.K. Feaster, A.G. Cadar, L. Wang, C.H. Williams, Y.W. Chun, J.E. Hempel, N. Bloodworth, W.D. Merryman, C.C. Lim, J.C. Wu, B.C. Knollmann, and C.C. Hong, Matrigel Mattress: A Method for the Generation of Single Contracting Human-Induced Pluripotent Stem Cell-Derived Cardiomyocytes. Circ Res 117 (2015) 995-1000.
[8] M. Paci, E. Passini, A. Klimas, S. Severi, J. Hyttinen, B. Rodriguez, and E. Entcheva, All-Optical Electrophysiology Refines Populations of In Silico Human iPSC-CMs for Drug Evaluation. Biophys J 118 (2020) 2596-2611.
[9] D. Shah, C. Prajapati, K. Penttinen, R.M. Cherian, J.T. Koivumaki, A. Alexanova, J. Hyttinen, and K. Aalto-Setala, hiPSC-Derived Cardiomyocyte Model of LQT2 Syndrome Derived from Asymptomatic and Symptomatic Mutation Carriers Reproduces Clinical Differences in Aggregates but Not in Single Cells. Cells 9 (2020).
[10] D. Shah, L. Virtanen, C. Prajapati, M. Kiamehr, J. Gullmets, G. West, J. Kreutzer, M. Pekkanen-Mattila, T. Helio, P. Kallio, P. Taimen, and K. Aalto-Setala, Modeling of LMNA-Related Dilated Cardiomyopathy Using Human Induced Pluripotent Stem Cells. Cells 8 (2019).
[11] E.M. Higgins, J.M. Bos, S.M. Dotzler, C.S. John Kim, and M.J. Ackerman, MRAS Variants Cause Cardiomyocyte Hypertrophy in Patient-Specific Induced Pluripotent Stem Cell-Derived Cardiomyocytes: Additional Evidence for MRAS as a Definitive Noonan Syndrome-Susceptibility Gene. Circ Genom Precis Med 12 (2019) e002648.
[12] A. Ahola, R.P. Polonen, K. Aalto-Setala, and J. Hyttinen, Simultaneous Measurement of Contraction and Calcium Transients in Stem Cell Derived Cardiomyocytes. Ann Biomed Eng 46 (2018) 148-158.
[13] P. Liang, K. Sallam, H. Wu, Y. Li, I. Itzhaki, P. Garg, Y. Zhang, V. Vermglinchan, F. Lan, M. Gu, T. Gong, Y. Zhuge, C. He, A.D. Ebert, V. Sanchez-Freire, J. Churko, S. Hu, A. Sharma, C.K. Lam, M.M. Scheinman, D.M. Bers, and J.C. Wu, Patient-Specific and Genome-Edited Induced Pluripotent Stem Cell-Derived Cardiomyocytes Elucidate Single-Cell Phenotype of Brugada Syndrome. J Am Coll Cardiol 68 (2016) 2086-2096.

Biology Open • Supplementary information
